# Comparative Study of the Prevalence of Intestinal Parasites in Low Socioeconomic Areas from South Chennai, India

**DOI:** 10.1155/2014/630968

**Published:** 2014-01-21

**Authors:** Jeevitha Dhanabal, Pradeep Pushparaj Selvadoss, Kanchana Muthuswamy

**Affiliations:** Department of Biochemistry, Sathyabama University Dental College & Hospitals, Rajiv Gandhi Salai, Sholinganallur, Chennai, Tamil Nadu 600119, India

## Abstract

Intestinal parasites cause one of the most important health problems through their effects in causing undernourishment morbidity and incapacitation due to their behavior particularly in children compared to adults. This study was intended to state the prevalence of intestinal parasites between the slum dwellers of different areas in south Chennai. Among the total of 256 samples collected between the ages of 0–50 yrs, 194 samples were positive. Standard laboratory techniques for parasitological diagnosis were carried out for each sample. *Entamoeba coli* (23%), *Cyclospora* sp. (22.2%), *Entamoeba histolytica* (21.8%), *Giardia intestinalis* (14.4%), *Ascaris lumbricoides* (6.2%), *Trichuris trichiura* (1.1%), and *Hymenolepis nana* (2.7%) were found in the dwellers of low socioeconomic areas. The data on the prevalence of parasites with respect to sex and age showed that the females harbored more numbers of parasites when compared to males. Further, with respect to age, children and teenagers had surplus parasites compared to old age groups. The percentage of educational status showed a reduction in the number of parasites in the higher education dwellers. These parasites could be prevented by possible grouping of better ecological design and hygiene. Conclusively, the examination of personal hygiene as well as routine medical examination and treatment is strongly recommended in the low socio-economic areas.

## 1. Introduction

Intestinal parasitic diseases constitute a global health burden in numerous developing countries mainly due to fecal contamination of water and food [1], sympathetic climatic, and environmental and sociocultural factors enhancing parasitic transmissions [2, 3]. These parasites dwell in the gastrointestinal tract in humans and other animals [4]. In urbanized countries, protozoan parasites commonly cause gastrointestinal infections in contrast to helminthes [5]. Amoebiasis is the third most important reason for death from parasitic diseases wide-reaching, with its furthermost impact on the people of developing countries. The World Health Organization (WHO) estimates that approximately 50 million people worldwide endure insidious amoebic infection each year, resulting in 40–100 thousand deaths yearly [6, 7]. Current estimates suggested that *Ascaris lumbricoides* can infect over a billion, *T. trichiura* can infect 795 million, and hookworms can infect 740 million people [8].

Intestinal helminths hardly ever cause death. As an alternative, the saddle of disease is related to less mortality than to the chronic and subtle effects on health and nutritional status of the host [9, 10]. In addition to their health effects, intestinal helminth infections also damage physical and mental development of children, prevent educational achievement, and hamper economic development [11, 12]. The common parasites that come upon in most of the preceding systematic investigations include *Ascaris lumbricoides*, hookworms (*Necator americanus*), *Trichuris trichiura*, *Strongyloides stercoralis*, *Entamoeba histolytica*, and *Giardia intestinalis *[13, 14]. These are reliant on poverty, miserable personal hygiene, piteous environmental care, inadequate health services, and lack of proper and necessary awareness of the transmission mechanisms and life-cycle patterns of these parasites [15–17].

Like other developing countries, intestinal parasitic infections are a major health problem in India. Studies conducted in children of rural and urban location in and around Chennai have reported the prevalence of intestinal parasitic diseases ranging from 60 to 91% [18] among which *Ascaris lumbricoides *was the most common helminthic parasite detected (52.8%) followed by *Trichuris trichiura* (45.6%). There is insufficiency of epidemiological data on the diffusion and prevalence of intestinal parasites in low socioeconomic people from south Chennai. This trend prompted us to evaluate the distribution of intestinal parasites among the slum dwellers in different areas of south Chennai by their age, education, nutrition, and hygienic factors.

## 2. Study Plan and Methods

### 2.1. Study Area

This study was conducted in main five low socioeconomic areas in south Chennai, namely, Thiruvanmiyur, Foreshore Estate, T. Nagar, Santhome, and Saidapet.

### 2.2. Study Design

The study was conducted between January and June of 2013 with the cooperation of local community, which possess the record for each member in the area. The fieldwork involved house-to-house visits, encouraging participation from each individual. Verbal informed consent was obtained from each individual before the study. Name, sex, age, education, nutrition, and family relationship details were collected. Fresh stool sample was collected and the individuals were also interviewed about their economic background, health status, toilet facilities, water facilities, child's nutritional status, local treatments, and previous parasitic infections.

### 2.3. Collection of Samples

Proper collection of sample is important for the detection and identification of intestinal parasites. A small screw capped plastic bottler with wooden scoop was provided to each person who was agreed to participate in the study. They were advised to fill half the bottle and discard the scoop after use. The next day samples were collected and brought to the laboratory for processing. All the containers along with specimen were properly labelled with the respective sample number, date, and area. Total of 256 samples were collected, from which 32, 63, 78, 49, and 34 samples were from age groups of 0–10 yrs, 10–20 yrs, 20–30 yrs, 30–40 yrs, and 40–50 yrs, respectively. Among those, 134 samples were from females and 122 samples were from males.

The following precautions were taken before collection of faeces. Consents were instructed not to mix urine with stool sample and also ensured that oil, oily emulsion, barium, or bismuth salts were not given before stool examination.

### 2.4. Preservation of Samples

Once the specimen was transported to the laboratory, saline, iodine wet mount, and other staining techniques were performed. The remaining specimen was preserved with 10% formalin and concentration techniques like sedimentation and floatation were performed. Preservation of faecal specimens is essential to maintain protozoal morphology and also to prevent further development of helminthic eggs and larvae.

### 2.5. Microscopic Examination-Staining Methods

The recognition of intestinal parasites was observed by using a binocular microscope under 10x and confirmed by observing under 40x [19–30].

### 2.6. Saline and Iodine Wet Mount

Approximate 2 mg of stool sample was picked up using a wooden stick and mixed with a drop of normal saline (0.9%) on a glass slide with applicator stick. If it was a formed stool, materials were taken from well inside the sample to look for parasite eggs. The preparation was covered with a cover slip and observed under the microscope. For iodine wet mount preparation, approximately 2 mg of stool sample was picked up using a wooden stick and mixed with a drop of dilute Lugol's iodine. It was covered with a coverslip and observed under the microscope.

### 2.7. Modified Ziehl-Neelsen Stain (Acid Fast Staining)

The smear on slide was fixed with methanol for 10 min and 5–7 drops of carbol fuchsin were flooded for 2-3 minutes. Then, it was decolourised with 5% sulphuric acid for 30 seconds. Then, the smear was counter-stained with methylene blue for a minute. Finally, the smear was rinsed, drained, air-dried, and examined under 10x, 40x, and oil immersion (100x).

### 2.8. Giemsa Stain

The smear on slide was fixed with methanol. Giemsa stain and buffer solution (1 : 10 ratio) was applied for 1 h. The smear was washed with buffer solution, allowed preparation for about 30 sec. The slide was blot-dried and observed under oil immersion (100x).

### 2.9. Floatation Techniques

1 mL of stool sample was mixed with few drops of salt solution and was stirred continuously to make as suspension. More salt solution was added to fill the container. Crude matter, which was floated, was removed. The container was placed on a level surface and the final filling of the glass container until a convex meniscus was formed. A glass slide was carefully laid on top of the container so that its center was in contact with the fluid. The preparation was allowed to stand for 20–30 min after which the glass slide was quickly lifted, turned over, smoothly so as to prevent spillage of the liquid, and examined under the microscope.

### 2.10. Zinc Sulphate Centrifugal Floatation

A fine stool suspension was made by mixing 1 g of stool and 10 mL of lukewarm distilled water. The coarse particles were removed by straining through a wire gauge. The filtrate was collected in a tube and centrifuged for 1 min at the rate of 2500 rpm. The supernatant fluid was poured off and distilled water was added to the sediment. It was shaken well and centrifuged and the procedure was repeated two to three times until the supernatant fluid became clear, which was then poured off. 3-4 mL of a 33% zinc sulphate was added to the sediment. The sediment was stirred and further zinc sulphate solution was added to fill the tube up to the top and centrifuged again for at least 1 min at 2500 rpm. The surface film was then removed by a loop on to a glass slide, covered by a cover slip, and observed under the microscope.

### 2.11. Formal-Ether Concentration

1 g of stool sample was fixed to emulsifying in 7 mL of 10% formal saline and kept for 10 min. It was then strained through a wire gauge and the filtrate was collected in a centrifuge tube. 3 mL of ether was added to it and the mixture was shaken vigorously for 1 min. It was then centrifuged at 2000 rpm for 2 min and then al-lowed to settle. The debris was loosened with a stick; the upper part of the test tube was cleared of fatty debris and the supernatant fluid was decanted, leaving 1 or 2 drops. The deposit, after shaking, was poured on to a glass slide, and a cover slip placed over it and the specimen was examined. This process was suitable for both protozoal cysts and helminthes eggs [31].

## 3. Results

**Figure 1:**
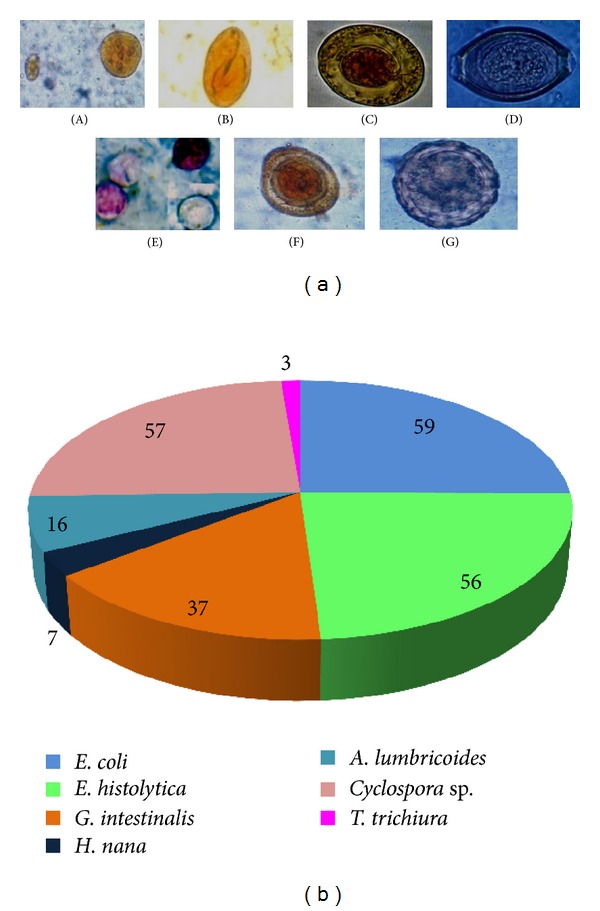
Parasites identified from stool samples. (a) Microscopic observation of protists and eggs (A) *Entamoeba coli;* (B) *Giardia intestinalis; *(C) *Hymenolepis nana; *(D)* Trichuris trichiura; *(E)* Cyclospora* sp. (F)* Ascaris lumbricoides*—decorticated egg; (G)* A. lumbricoides*—corticated egg. (b) Distribution of parasites in pie diagram.

Figure 1(a) depicts the various intestinal parasites which were observed under microscope. Each stool sample collected from apparently healthy people was processed for intestinal parasites using various parasitological methods. The distributions of the parasites were well described by the positive cases. *Entamoeba coli* was found to be the most predominant parasite in the human communities with 59 (23%) positive cases. And the second most predominant parasite was *Cyclospora* sp. with 57 (22.2%) positive cases. Among the other protozoan parasites, *E. histolytica* was found to be predominant with 56 (21.8%) positive cases followed by *Giardia intestinalis* with 37 (14.4%) positive cases. In the case of helminthes, *Ascaris lumbricoides* was predominant with 16 (6.2%) positive cases followed by *Trichuris trichiura* with 3 (1.1%) positive cases. Finally, the metazoal parasite *Hymenolepis nana* was found predominant with 7 (2.7%) positive cases (Figure 1(b)).

**Figure 2:**
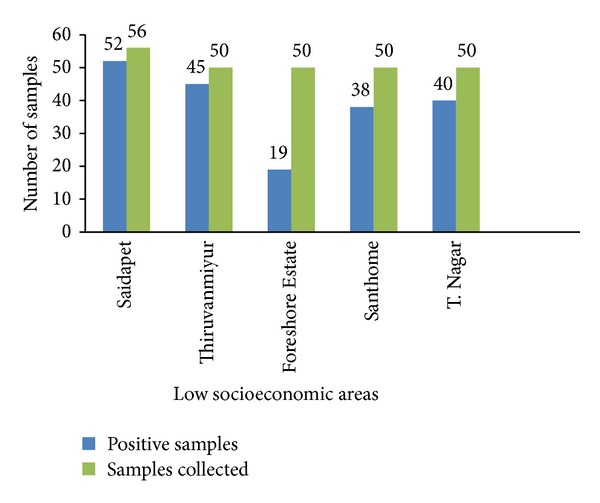
Distribution of samples in low socioeconomic areas from south Chennai.

Figure 2 depicts the distribution of positive and negative cases in low socioeconomic area of south Chennai. The total samples collected from Saidapet slum dwellers was 56, of which 52 (92.8%) were positive. 50 samples were collected from Thiruvanmiyur, of which 45 (90%) were positive. 19 (38%) samples were positive from 50 samples which was processed from Foreshore Estate dwellers. Out of the 50 samples from Santhome dwellers, 38 (76%) were positive, and out of the 50 samples from T. Nagar dwellers, 40 (80%) were positive.

**Figure 3:**
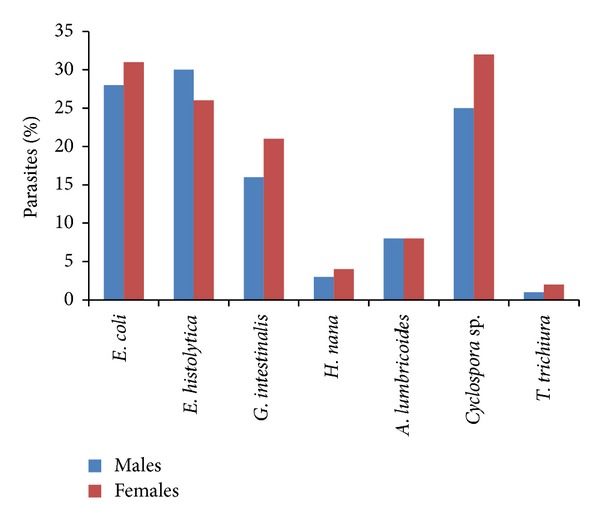
Distribution of parasites among males and females of low socioeconomic areas.

Figure 3 depicts the percentage result on the prevalence of parasites in male and female. This result showed that females harboured larger proportion of infections in contrast to the males who frequently carry out moderate infections. *E. coli* showed 28% in males whereas in females it's showed 31%. Followed by *E. histolytica* (30%), *G. intestinalis* (16%), *H. nana* (3%), *Trichuris trichiura* (1%), and *Cyclospora* sp. (25%) in males but in females there was a slight difference in number follows *E. histolytica* (26%), *G. intestinalis* (21%), *H. nana* (4%), *Trichuris trichiura* (2%), and *Cyclospora* sp. (32%). *Ascaris lumbricoides* (8%) was similar in males and females. *E. coli, G. intestinalis,* and *Cyclospora* sp. in particular were found to be of more numbers in females than in males.

**Figure 4:**
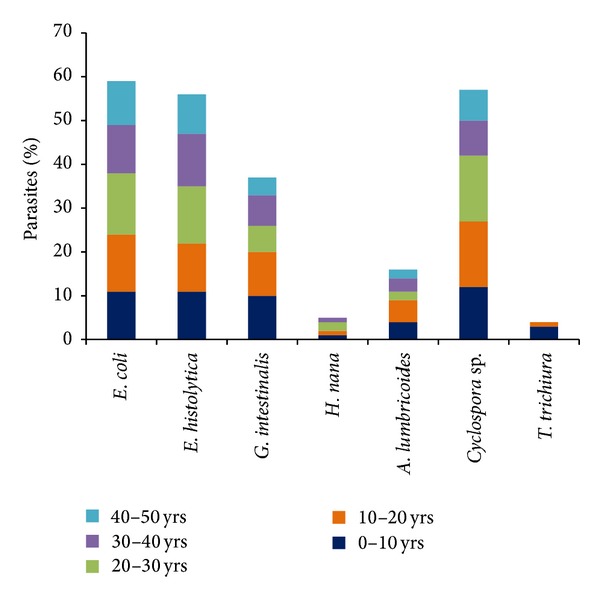
Distribution of parasites among different age groups from low socioeconomic areas from south Chennai.

Figure 4 depicts the age prevalence profile of the intestinal parasites. The children and teenagers were found to harbour increased numbers of parasites in comparison with old age group. *E. coli *(11.8%), *E. histolytica *(11.2%), and *Cyclospora* sp. (11.4%) were found aggregated in all age groups. *Trichuris trichiura* was completely absent in all the other age groups except 3% in 0–10 yrs and 1% in 10–20 yrs. *Giardia intestinalis *(7.4%) and* Ascaris lumbricoides *(3.2%) showed a decreasing profile with the increasing age group.

**Figure 5:**
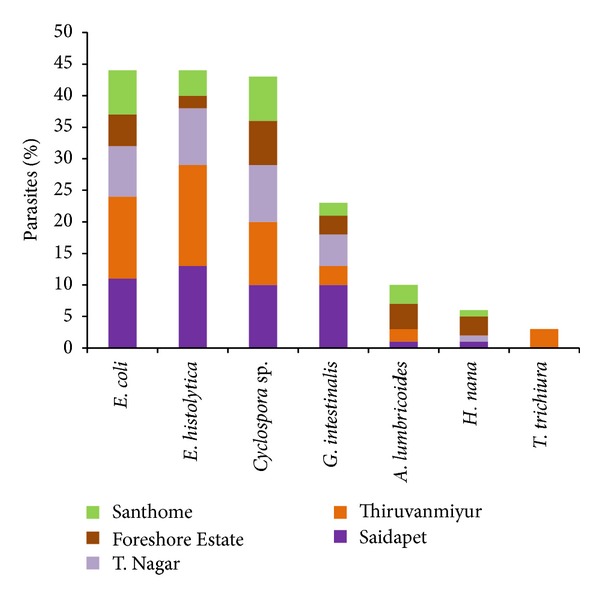
Distribution of parasites among different areas from south Chennai.

Figure 5 provides information on the distribution of the parasites in different areas. Saidapet and Thiruvanmiyur dwellers had an increasing proportion of *E. histolytica *(13 and 16%), *Entamoeba coli *(11 and 13%)*, Giardia intestinalis *(10 and 3%), and* Cyclospora* sp. (10 and 10%) when compared to *Hymenolepis nana *(1%). *T. trichiura *(3%) was found only among Thiruvanmiyur dwellers. *Entamoeba coli *(7%) and *Cyclospora* sp. (7%) were found equally predominant in Santhome dwellers. T. Nagar and Foreshore Estate dwellers had less number of parasites compared to Saidapet and Thiruvanmiyur dwellers. *E. coli* and *E. histolytica* were found in large numbers and evenly distributed in all areas.

**Figure 6:**
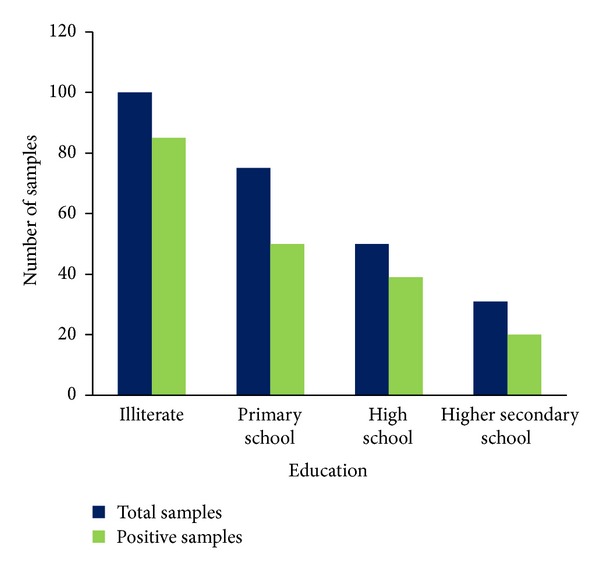
Education status of the low socioeconomic dwellers from south Chennai.

The educational status of the dwellers was as follows, 85% of dwellers were illiterate, 66.6% was primary school educators, 78% was high school educators, and 64.5% was higher secondary educators (Figure 6). The percentage of positive cases shows a decrease with regard to increase in educational status. A large number of positive cases were found completely in illiterate population. This shows the implications of education in sanitary living.

## 4. Discussion

Roughly half of the world's population lives under the conditions that generate nutritional stress and parasitic diseases with protozoan parasites or helminthes. The present study included a parasitological analysis of 256 stool samples in low socioeconomic areas of south Chennai with special attention to both intestinal protozoa and helminthes. This study has documented a high prevalence (75.7%) of intestinal parasites in the dwellers from south Chennai, India. Saidapet and Thiruvanmiyur dwellers were found to harbor the maximum number of positive cases. The latrines in these two areas were found to be crude and difficult to clean. Less number of positive cases was found to be in Foreshore Estate area when compared to other areas. T. Nagar and Santhome areas almost had equal number of positive cases. Inadequate sanitary measures and problems of drainage may contribute to this high prevalence of parasitic infection. In addition, good hand washing which ordinarily should interrupt the transmission of some of the parasites is expectedly inadequate in situations where water supply takes a lot of manual effort and the tendency is to use water sparingly. This eventually results in further transmission by direct and indirect contact. The common practice of emptying the water portion of filled septic tanks into the gutters and burying the faecal wastes in the soil might has also contributed to the high prevalence of intestinal parasites. The watery portion eventually contaminates bodies of water used by humans and the buried wastes contaminate underground surface water. This might contribute the epidemiology of the intestinal parasites in the slum areas in this part of the city.

The high prevalence rate of *Entamoeba* species (44.9%) showed that the infection transfer between persons through food or water is high and this proves that there is high level of contamination by human faeces [18]. In some areas, the water pipes are passed through drainage pathways and, in some instances, the pipes are broken and left unattended, which eventually contaminate the drinking water which thereby led to the occurrence of the emerging protozoan pathogen *Cyclospora* [32], responsible for severe diarrhoea. Secondly, the comparatively high occurrence of *Cyclospora* sp. in apparently healthy populations proves the elevated levels of morbidity and mortality linked with them when the immune system is compromised. Certain ranges of temperature, humidity, and other environmental factors permit or assist sporulation and endurance of oocysts. This partly explains the noticeable seasonality of the Cyclospora infection observed in this study and might be different in diverse settings [33, 34]. Hence, regular steps should be taken to become aware of these parasites.

In this study, presence of *G. intestinalis* was 14.4% whereas the same was reported by Laila in the stool samples of the people in Jordan [35]. Researchers state that, amongst the nematodes, the most common parasite isolated was *Ascaris *[36] followed by *Trichuris, *a major parasite in the age group of 0–10 yrs that was conventional in their study carried out [18–24]. It is to be noted in this study that the people of the age group (10–20 yrs) were highly infected with intestinal parasite (except *Trichuris*), thus confirming almost equal chances of exposure especially by faeco-oral route due to the high road side activity.

The animals such as cattle, sheep, and goat may serve as incidence and numbers of patients with prolonged diarrhoea indicating the need for increased clinical vigilance with regard to the inclusion of these coccidian parasites in the routine screening for intestinal parasites, especially if the patient is immunocompromised. In relation to gender, there was a considerable difference in the prevalence of the intestinal parasites. The infections are likely to be linked to the everyday activities of the individuals rather than gender. Efforts should be intensified towards the stipulation of sufficient clean water and public tutoring on enhanced personal and ecological hygiene as it will go to an extensive way in plummeting the morbidity and mortality associated with intestinal parasitism.

## 5. Conclusion

This study has categorized the high predominance rate of intestinal parasites in low socioeconomic areas from south Chennai. On the subject of 256 samples collected, 194 samples were observed to be positive cases. In the age prevalence profile, children's and teenagers were found to harbor more numbers of parasites in comparison with old age group. Saidapet and Thiruvanmiyur areas were found to harbour maximum number of positive cases. The dispersed distribution might reflect the heterogeneity in social behavioral and special factors affecting the degree of express of infection. Appropriate and detailed control method of sanitation must be considerably improved and applied at both the household and community levels. Further research has to be carried out to determine more quantitatively the public health significance of these intestinal parasites.
